# Psychosocial Predictors of Healthcare Decision-Making Capacity in Older Adults: The Role of Cognitive Functioning and Affective Symptoms

**DOI:** 10.3390/healthcare14172781

**Published:** 2026-09-01

**Authors:** Emília Santos, Ana S. Amaral, Rosa M. Afonso

**Affiliations:** 1 Department of Psychology and Education, University of Beira Interior, R. José Caetano Júnior, 6200-209 Covilhã, Portugal; emilia.santos@ubi.pt; 2 RISE-Health, Faculty of Health Sciences, University of Beira Interior, Av. Infante D. Henrique, 6200-506 Covilhã, Portugal

**Keywords:** capacity for decision-making, aging, depression, anxiety, cognitive functioning, older adults

## Abstract

**Background/Objectives**: The capacity for decision-making in healthcare is a critical component of autonomy in later life; however, its psychosocial determinants remain insufficiently understood. This study aimed to examine the relationship between decision-making capacity in healthcare, cognitive functioning, depressive and anxiety symptoms, and sociodemographic factors in cognitively healthy older adults, and to identify psychosocial predictors of capacity for decisions. **Methods**: A cross-sectional study was conducted with 60 older adults. Participants completed measures for decision-making capacity in healthcare, cognitive performance, depressive symptoms, and anxiety symptoms. Spearman correlations and multiple linear regression analyses were performed. Regression assumptions were formally tested, and sensitivity analyses were conducted. **Results**: The capacity decision-making in healthcare was positively associated with cognitive performance but not significantly correlated with depressive or anxiety symptoms. Regression analyses revealed that age and depressive symptomatology were significant negative predictors, with the model explaining 44.9% of the variance (adjusted *R*^2^ = 0.375). Sensitivity analyses indicated that the age effect was stable across all specifications, whereas the contribution of depressive symptomatology was concentrated among a few participants with clinically elevated symptoms. Anxiety symptoms were not significant predictors. A strong positive association was observed between depressive and anxiety symptoms. **Conclusions**: These findings support a multidimensional model of the capacity for decision-making in healthcare, highlighting the central role of cognitive functioning and age, and suggesting that depressive symptoms may become clinically relevant at higher symptom levels, even in cognitively healthy older adults. The results underscore the importance of integrating cognitive and emotional assessment in capacity evaluations and suggest that addressing depressive symptoms may contribute to preserving decisional autonomy. Future research should adopt longitudinal and clinically diverse samples to further elucidate these relationships.

## 1. Introduction

The capacity for taking decisions in healthcare is a fundamental component of clinical practice, particularly in situations where individuals may be unable to make informed decisions independently [1]. Decision-making in healthcare refers to a cognitive and emotional ability to evaluate information, consider alternatives, and accept or refuse proposed medical interventions [2]. This process encompasses decisions regarding diagnostic procedures, treatment options, and other healthcare interventions, requiring the integration of specific cognitive functions [3].

Assessing the capacity for decision-making is especially relevant in later life, as aging is frequently associated with increased health problems and cognitive decline, leading to a higher demand for formal capacity evaluations [4]. Older adults with cognitive impairment tend to make less advantageous decisions and exhibit more dysfunctional decision-making profiles compared to cognitively healthy individuals, given that decision-making relies on multiple cognitive domains, including semantic and episodic memory as well as executive functioning [5].

Evaluating the capacity for decision-making in healthcare in older adults is inherently complex, requiring the consideration of psychological variables and contextual factors [6]. This complexity is heightened in conditions such as mild cognitive impairment and early-stage dementia, where deficits may be subtle, yet clinically significant [6]. A single clinical interview is typically insufficient; comprehensive assessments should integrate psychological, functional, and neuropsychological evaluations, alongside medical record reviews and collateral information, to ensure a thorough understanding of the individual’s abilities [7].

A widely accepted framework for assessing the capacity for decision-making is the four-abilities model proposed by Grisso and Appelbaum [8], which conceptualizes capacity as comprising understanding, appreciation, reasoning, and expression of choice. Understanding refers to the ability to comprehend relevant clinical information, including diagnoses, prognoses, treatment options, and associated risks and benefits [9]. Individuals must demonstrate meaningful comprehension rather than mere repetition of information [10]. Appreciation involves the ability to apply this information to one’s own situation. Reasoning reflects the capacity to logically manipulate information and weigh alternatives, while expression of choice refers to the ability to communicate a clear and consistent decision [10]. Impairment in any of these domains may compromise autonomy and result in diminished decisional capacity [11].

Effective decision-making has traditionally been conceptualized as a process guided by rational evaluation, leading to choices that maximize personal benefit [12]. However, this perspective has evolved to recognize that decision-making is not solely a cognitive process but rather emerges from the interaction between cognitive, emotional, and motivational factors [1]. In this context, affective states—particularly depression and anxiety—play a critical role in shaping how individuals process information and evaluate alternatives [12,13,14].

Depressive symptomatology has been consistently associated with impairments in decision-making. Older adults experiencing active depression demonstrate less adaptive decision-making profiles compared to those in remission or without depressive symptoms [14]. Depression may negatively impact decisional capacity primarily by affecting appreciation—the ability to recognize and value the consequences of different options—while also influencing understanding and reasoning to a lesser extent [11]. Empirical evidence suggests that individuals with higher levels of depressive symptoms tend to seek less information, underutilize available resources, and show reduced ability to resolve ambiguity, even when decision-support tools are provided [12]. Additionally, avoidance of anxiety-provoking situations may lead to the rejection of potentially beneficial but uncertain options [12].

From a cognitive perspective, non-depressed individuals tend to exhibit a positive processing bias, attending more to favorable information and recalling positive experiences more readily [15]. This bias can have adaptive value by promoting motivation and persistence. In contrast, the absence of such bias and the presence of negative cognitive schemas in depression may impair evaluative processes essential for sound decision-making, reinforcing the need for ethically grounded and methodologically robust capacity assessments [11].

Anxiety disorders, among the most prevalent psychiatric conditions globally, are characterized by excessive fear, persistent worry, and avoidance behaviors [16,17]. In older adults, anxiety is associated with functional impairment, cognitive deficits—particularly in memory—and poorer outcomes in comorbid medical conditions [17]. Moreover, even mild worry symptoms may help predict early cognitive decline in healthy, community-dwelling older adults, underscoring the role of subtle emotional manifestations as relevant indicators of cognitive change [18]. Although anxiety is known to influence everyday decision-making, often by promoting threat avoidance [13], its specific impact on the capacity for taking decisions in healthcare remains underexplored, highlighting an important gap in the literature.

Mental health conditions are highly prevalent among older adults, with approximately 14% of individuals aged 60 years and older experiencing a mental disorder, most commonly depression [19,20]. Despite not being a normative aspect of aging, depression is frequently underdiagnosed and undertreated, contributing to adverse outcomes such as functional decline, poorer physical health, and increased mortality [21].

Depression and anxiety frequently co-occur and, although distinct in symptomatology, both are associated with significant impairments in decision-making [5,22]. These impairments are particularly relevant in clinical contexts, as they may compromise an individual’s ability to make autonomous and informed healthcare-related decisions [11]. Alterations in reward and threat processing have been proposed as underlying mechanisms, influencing how individuals evaluate potential gains and losses [22].

Despite the growing body of research on the capacity for decision-making in healthcare, most studies have predominantly focused on cognitive determinants, while comparatively less attention has been given to psychosocial factors, particularly affective symptoms such as depression and anxiety, and their differential contribution to decisional abilities [11,12]. Evidence suggests that the magnitude of these effects varies depending on symptom severity and population characteristics [11]. Although both depression and anxiety have been associated with impairments in decision-making, the specific mechanisms through which these conditions influence distinct components of the capacity for decisions in older adults remain insufficiently understood [13,22]. Although cognitive functioning and emotional well-being are interrelated, change in one domain does not consistently predict change in the other in older adults; instead, factors such as education, physical functioning, and self-rated health emerge as key predictors of both cognitive trajectories and depressive symptoms [23]. The existing literature is limited by a relative scarcity of studies examining these associations in populations with varying levels of cognitive functioning [6].

Moreover, few studies have adopted a multidimensional approach that simultaneously considers cognitive and emotional variables when examining predictors of capacity for decision-making in healthcare. As such, a more integrative perspective is needed to better understand the relative and combined influence of these factors on the capacity for decisions.

The present study addresses these gaps by examining the capacity for decision-making in healthcare within a multidimensional framework, incorporating cognitive functioning and affective symptomatology (depression and anxiety) in a sample of older adults. By exploring psychosocial predictors of decisional capacity, this study aims to contribute to a more comprehensive understanding of the factors that support or undermine autonomy in healthcare contexts. Ultimately, these findings may inform the development of more accurate assessment practices and targeted interventions to promote person-centered and ethically grounded care in aging populations.

The present study aims to: (1) assess the capacity for making decisions in healthcare, cognitive functioning, and depressive and anxiety symptoms in older adults; (2) examine the relationship between the capacity for decision-making and depressive and anxiety symptomatology; (3) analyze the association between cognitive functioning and the capacity for decision-making; and (4) explore psychosocial predictors of capacity for decision-making in healthcare.

## 2. Materials and Methods

### 2.1. Participants

A total of 60 community-dwelling older adults aged 60 years and above participated in this study (*M* = 71.17, *SD* = 5.96). The majority were female (*n* = 45.75%), married (*n* = 39, 65%), and retired (*n* = 52, 86.7%). Regarding education, 31.7% (*n* = 19) had completed secondary education, and most participants resided in predominantly urban areas (*n* = 38, 63.3%).

The functional status was assessed using the IAFAI (Inventário de Avaliação Funcional de Adultos e Idosos [Functional Assessment Inventory for Adults and Older Adults]), indicating overall low levels of functional impairment. Mean disability scores were 1.24% (*SD* = 2.17) for basic activities of daily living (ADL), 0.41% (*SD* = 1.28) for instrumental ADL (family-related), and 0.07% (*SD* = 0.36) for advanced instrumental ADL. The global functional disability index averaged 1.71% (*SD* = 2.75). Hypertension was the most frequently reported medical condition (*n* = 15, 25%).

### 2.2. Instruments

#### 2.2.1. Capacity Assessment Instrument—Health (CAI-Health)

The capacity for decision-making was assessed using the Capacity Assessment Instrument—Health (CAI-Health) [3,24]. This instrument includes a clinical vignette (gender-matched) describing a hypothetical case of knee osteoarthritis, followed by a structured interview comprising 19 items for assessing four core abilities: understanding, appreciation, reasoning, and expression of choice. Responses are scored on a 3-point scale (0–2), with higher scores indicating better decisional capacity.

#### 2.2.2. Cognitive Functioning

Cognitive performance was assessed using the Montreal Cognitive Assessment (MoCA) [25,26], a widely used screening tool with a maximum score of 30 points. It evaluates multiple cognitive domains, including executive functions, visuospatial abilities, memory, attention, language, and orientation. Portuguese normative data adjusted for age and education were considered [27].

#### 2.2.3. Anxiety Symptoms

Anxiety was assessed using the Geriatric Anxiety Inventory (GAI) [28,29], a 20-item self-report instrument specifically developed for older adults. The Portuguese version demonstrates good psychometric properties, with a cut-off score of 10/11 (sensitivity = 0.900; specificity = 0.859).

#### 2.2.4. Depressive Symptoms

Depressive symptoms were measured using the Geriatric Depression Scale (GDS-30) [30,31,32], a 30-item dichotomous self-report scale. Scores are categorized as follows: ≤10 (no clinically significant symptoms), 11–20 (mild depression), and >20 (severe depression) [33].

#### 2.2.5. Sociodemographic and Clinical Data

A structured questionnaire was used to collect information on age, gender, education, marital status, residence, and occupational status, as well as self-reported chronic conditions based on national prevalence data [34].

### 2.3. Procedures

This study was conducted within the framework of the project “Assessment of Healthcare Decision-Making Capacity—Development and Validation of the IACTD-CS”, approved by the Ethics Committee of the University of Beira Interior (CE-UBI-Pj-2020-072).

Participants were recruited using community-based strategies, including dissemination of an informational poster via social media and a Microsoft Forms link, as well as through formal partnerships with institutions working directly with older adult populations. These strategies aimed to maximize outreach and facilitate access to potential participants.

Individuals who expressed interest in participating were provided with detailed information regarding the study’s objectives, procedures, and implications. Written informed consent was obtained prior to participation, ensuring adherence to ethical principles of voluntariness, confidentiality, and anonymity.

Data were collected through face-to-face structured interviews conducted by four trained interviewers, with an average duration of approximately 45 min. Additional participants were recruited beyond those identified through institutional contacts to ensure adequate sample size.

Inclusion criteria were: (a) age ≥ 60 years; (b) at least 1 year of schooling; (c) Portuguese as a native language; (d) residence in the community; and (e) absence of cognitive impairment, as determined by the MoCA. A total of 74 individuals were initially assessed, of whom 14 were excluded due to evidence of cognitive impairment, resulting in a final sample of 60 participants.

### 2.4. Data Analysis

Data were analyzed using IBM SPSS Statistics (version 30). The final dataset included 60 participants who met all inclusion and exclusion criteria. Descriptive statistics (means, standard deviations, frequencies, and percentages) were computed to characterize the sample and to describe levels of depressive and anxiety symptoms. There were no missing data. Because all instruments were administered in face-to-face interviews, complete responses were obtained from every participant on every measure, and no case was excluded on this basis.

The distribution of continuous variables was assessed using the Kolmogorov–Smirnov test, together with inspection of skewness and kurtosis. Depressive symptoms (skewness = 1.96, kurtosis = 4.05) and anxiety symptoms (skewness = 1.22, kurtosis = 0.52) showed marked positive skew with a pronounced floor effect, 47% of the sample being concentrated in the three lowest scores of the GDS-30. Because the Pearson coefficient is not resistant to the leverage exerted by isolated observations in the upper tail of such distributions, Spearman’s rank-order correlation coefficients were used to examine associations between the capacity for decision-making in healthcare (CAI-Health), depressive symptoms (GDS), anxiety symptoms (GAI), and cognitive performance (MoCA). Correlation strength was interpreted based on conventional thresholds [35]. Internal consistency in the present sample was estimated using Cronbach’s α.

To examine predictors of capacity for decision-making in healthcare, a multiple linear regression analysis was performed [36]. The dependent variable was the capacity for decision-making (CAI-Health), and independent variables included age, education, cognitive performance (MoCA), depressive symptoms (GDS), and anxiety symptoms (GAI), selected based on theoretical and empirical evidence. Education was dummy-coded into three categories, with primary education (1st and 2nd levels of basic education; *n* = 17) as the reference category; the remaining categories were the 3rd level of basic education (*n* = 12), secondary education (*n* = 19), and higher education (*n* = 12). Statistical significance was set at *p* < 0.05 for all analyses.

The assumptions of multiple linear regression were formally assessed [36]. Independence of residuals was examined with the Durbin–Watson statistic, homoscedasticity with the Breusch–Pagan test, functional form with Ramsey’s RESET test, normality of residuals with the Shapiro–Wilk test, and multicollinearity with variance inflation factors (VIF) and tolerance. Influence was evaluated through standardized residuals, leverage values, and Cook’s distance, applying the conventional thresholds of |SR| > 2, *h* > 2(*k* + 1)/*n*, and *D* > 4/*n*. Effect sizes were quantified as squared semi-partial correlations (*sr*^2^) and Cohen’s *f*^2^, and regression coefficients were additionally interpreted in the original metric of the instruments.

Given the ratio of cases to predictors, sensitivity analyses were conducted. A reduced model was estimated in which the three education indicators were removed: education was included as a sociodemographic covariate rather than as one of the constructs specified in the study aims, and its three dummy variables consumed three degrees of freedom. Consistent with this, the education block did not contribute to the model, *F*(3, 52) = 1.375, *p* = 0.261. All four predictors corresponding to the study aims—age, cognitive performance, depressive symptoms, and anxiety symptoms—were retained, with relevance to the latter, which had not reached significance in the full model. No predictor was removed based on its statistical significance. Both the full and the reduced models were additionally re-estimated after exclusion of the cases identified as influential. A sensitivity power analysis was also performed. With *N* = 60, α = 0.05, and seven predictors, the design afforded 80% power to detect an effect of *f*^2^ = 0.273 (equivalent to *R*^2^ = 0.214); detection of a medium effect (*f*^2^ = 0.15) would have required *N* = 103. The resulting ratio of 8.6 cases per predictor falls below conventional recommendations, which motivated the reduced model analysis reported below.

## 3. Results

Descriptive statistics for the study variables are presented in Table 1. Participants showed a mean score of 27.10 (*SD* = 4.16) in the capacity for decision-making in healthcare, with scores ranging from 15 to 34. Mean depressive symptomatology was 5.47 (*SD* = 5.16; range = 0–24), while anxiety symptoms presented a mean of 5.37 (*SD* = 5.77; range = 0–20). Cognitive performance, assessed with the MoCA, yielded a mean score of 23.65 (*SD* = 2.84), ranging from 17 to 30.

Given the skewness of the affective measures, medians and interquartile ranges are also reported: CAI-Health *Mdn* = 28.0 (*IQR* = 5.0), MoCA *Mdn* = 23.5 (*IQR* = 4.0), GAI *Mdn* = 4.0 (*IQR* = 7.0), and GDS *Mdn* = 3.0 (*IQR* = 5.2).

Internal consistency of the CAI-Health in the present sample was acceptable, Cronbach’s α = 0.730, with corrected item–total correlations ranging from 0.119 to 0.606.

Spearman correlation analyses (Table 2) indicated that the capacity for decision-making in healthcare was not significantly associated with depressive symptoms (*r_s_* = −0.076, *p* = 0.565) or anxiety symptoms (*r_s_* = −0.007, *p* = 0.958). However, a moderate, positive, and statistically significant correlation was found between decision-making capacity and cognitive performance (*r_s_* = 0.480, *p* < 0.001), suggesting that higher cognitive functioning is associated with better capacity for decisions.

A strong and statistically significant positive correlation was observed between depressive and anxiety symptoms (*r_s_* = 0.730, *p* < 0.001), indicating substantial co-occurrence of these affective symptoms in the sample. No significant associations were found between cognitive performance and depressive symptoms (*r_s_* = −0.108, *p* = 0.410) or anxiety symptoms (*r_s_* = −0.095, *p* = 0.468).

A multiple linear regression analysis was conducted to examine predictors of the capacity for decision-making in healthcare (Table 3). The model, which included age, education, cognitive performance, depressive symptoms, and anxiety symptoms, was statistically significant, *F*(7, 52) = 6.053, *p* < 0.001, explaining 44.9% of the variance (*R*^2^ = 0.449, adjusted *R*^2^ = 0.375). Multicollinearity remained within acceptable limits, although the two affective measures showed the highest values (GDS: VIF = 3.59, tolerance = 0.279; GAI: VIF = 3.55, tolerance = 0.281), reflecting their strong intercorrelation; all remaining VIF values were below 1.9.

Within the model, depressive symptomatology (β = −0.411, *p* = 0.040) and age (β = −0.490, *p* < 0.001) emerged as significant predictors of the capacity for decision-making. These findings indicate that higher levels of depressive symptoms and older age are associated with lower capacity for decision-making in healthcare. In contrast, anxiety symptoms, cognitive performance, and education did not significantly contribute to the model (Table 3). In terms of unique variance, age accounted for *sr*^2^ = 0.183 (*f*^2^ = 0.33) and depressive symptomatology for *sr*^2^ = 0.047 (*f*^2^ = 0.09). Expressed in the original metric, a 10-year difference in age corresponded to a difference of 3.4 points on the CAI-Health (0.82 *SD*), and a 10-point difference on the GDS-30 to a difference of 3.3 points (0.80 *SD*).

Assumption checks supported the model with one exception. Residuals were independent (Durbin–Watson = 2.06), homoscedastic (Breusch–Pagan *LM* = 4.28, *p* = 0.747), and consistent with a linear functional form (RESET *F* = 0.54, *p* = 0.585), but departed from normality (Shapiro–Wilk *W* = 0.927, *p* = 0.001; skewness = −0.93). The departure was moderate and, given the sample size, the parametric inference is likely to remain reasonably robust; the coefficient estimates are nonetheless interpreted below in the light of the influence diagnostics and sensitivity analyses rather than based on the nominal *p* values alone.

Influence diagnostics identified one participant exceeding all three conventional thresholds (standardized residual = −2.30, leverage = 0.306, Cook’s *D* = 0.293), the next largest Cook’s distance in the sample being 0.146. Two further participants showed high leverage (0.298 and 0.235) without discrepant residuals, indicating that they anchored rather than distorted the fitted slope. These three participants were the only ones with GDS-30 scores above 15 (21, 23, and 24) and were simultaneously at the ceiling of the GAI-20. Five cases (8.3%) had absolute standardized residuals above 2, close to the proportion expected under the model, and none exceeded 3.0 (range −2.96 to 1.93); no case exceeded the classical Cook’s distance threshold of 1.0. The three cases were retained in the primary analysis as clinically plausible observations rather than measurement errors.

The main sensitivity analyses are summarized in Table 4. The age coefficient was stable across every specification, in the reduced model (*B* = −0.323, *p* < 0.001) and after exclusion of the influential cases in both the full model (*B* = −0.290, *p* = 0.001) and the reduced model (*B* = −0.282, *p* = 0.001). The coefficient for depressive symptomatology was not stable: it retained significance in the reduced model (*B* = −0.390, *p* = 0.015) but was eliminated whenever the three participants with GDS-30 scores of 20 or above were excluded (*B* = +0.012, *p* = 0.951 in the full model; *B* = −0.086, *p* = 0.665 in the reduced model). Comparison of nested models further showed that the standardized coefficient of depressive symptoms increased in magnitude when anxiety symptoms were entered, from β = −0.290 in a model containing depression alone to β = −0.571 when both affective measures were included, a pattern consistent with classical suppression.

In the reduced model, cognitive performance (*B* = 0.223, *SE* = 0.169, β = 0.152, *p* = 0.192) and anxiety symptoms (*B* = 0.146, *SE* = 0.139, β = 0.203, *p* = 0.299) remained non-significant, as in the full model; *R*^2^ = 0.405, adjusted *R*^2^ = 0.362, *F*(4, 55) = 9.37, *p* < 0.001. Multicollinearity was comparable to that of the full model (GDS: VIF = 3.45; GAI: VIF = 3.46; age: VIF = 1.24; MoCA: VIF = 1.23). The reduced model was estimated to establish whether the conclusions depended on the number of predictors relative to the sample size, rather than to maximize explained variance.

## 4. Discussion

The present study aimed to examine the relationship between the capacity for decision-making in healthcare, cognitive performance, depressive and anxiety symptoms, and sociodemographic variables in cognitively healthy older adults. Overall, the findings support the multifactorial nature of decision-making capacity, highlighting the role of cognitive functioning and depressive symptomatology, while also revealing nuances that contribute to the current literature.

Consistent with prior research (e.g., [1,37]), a significant association was found between the capacity for decision-making in healthcare and cognitive performance. This finding reinforces the well-established notion that decision-making relies on multiple cognitive processes, including working memory, attention, and reasoning. Previous studies have shown that older adults with cognitive impairment are more likely to exhibit difficulties in decision-making compared to cognitively intact individuals [5,14]. The present results extend this evidence by demonstrating that even within a cognitively preserved sample, variability in cognitive functioning remains a relevant factor influencing decisional capacity. These findings underscore the importance of incorporating cognitive assessment into evaluations of decision-making capacity and support interventions aimed at maintaining or enhancing cognitive functioning as a means of promoting autonomy in later life.

Contrary to expectations, no significant associations were observed between the capacity for decision-making in healthcare and depressive or anxiety symptoms. This finding diverges from previous literature suggesting that affective states may impair decision-making processes, particularly through their impact on appreciation and reasoning abilities [11,38]. One possible explanation is that the relatively low levels of depressive and anxiety symptoms observed in this sample may not have reached a threshold sufficient to produce measurable impairments in decisional capacity. Additionally, it is plausible that the influence of affective symptoms operates indirectly, potentially mediated or moderated by cognitive functioning or other psychosocial variables.

It should be noted that the regression analyses revealed that depressive symptomatology emerged as a significant negative predictor of decision-making capacity when controlling for other variables. Since this contrasted with the non-significant bivariate correlation, the discrepancy was examined formally. As reported above, comparison of nested models showed that the standardized coefficient of depressive symptoms increased substantially in magnitude once anxiety symptoms were entered. This pattern characterizes classical suppression, and the two affective measures were strongly intercorrelated (Table 2), whereas anxiety symptoms showed no association with decisional capacity. As such, entering anxiety into the model removed from the depression measure variance that was unrelated to the criterion. Influence diagnostics, however, indicated that this pattern was concentrated in three participants who were simultaneously at the upper extreme of the GDS-30 and at the ceiling of the GAI-20, and the depression coefficient was eliminated when these cases were excluded. The association between depressive symptomatology and decisional capacity observed here is therefore confined to the upper tail of a distribution with a pronounced floor effect. It is compatible with a threshold effect, whereby depressive symptoms would bear on decisional capacity only once they reach clinical intensity, but the present design cannot distinguish such an effect from the influence of a small number of observations. Therefore, this finding is interpreted as preliminary and hypothesis-generating rather than as evidence that depressive symptoms independently predict decisional capacity in cognitively healthy older adults. Should such a threshold effect be confirmed in samples with greater symptom variability, it would align with theoretical perspectives emphasizing the interplay between cognitive and emotional processes in decision-making [1]. From a cognitive model standpoint, depressive symptomatology may be associated with decision-making through the activation of negative cognitive schemas, as described in Beck’s cognitive triad [39,40], which may lead to pessimistic evaluations of available options and reduced motivation to engage in information-seeking behaviors. This interpretation is consistent with empirical findings indicating that individuals with higher depressive symptoms tend to adopt less effective decision-making strategies and show reduced engagement with available resources [12].

In contrast, anxiety symptoms were not significantly associated with the capacity for decision-making in either correlational or regression analyses. Although previous studies suggest that anxiety may bias decision-making toward threat avoidance and risk aversion [12,13], the lack of significant findings in the present study may again be attributable to the low severity of symptoms in the sample or to the possibility that anxiety exerts more subtle or context-dependent effects that are not captured by global measures of decisional capacity. This finding highlights the need for further research examining the specific mechanisms through which anxiety influences different components of decision-making.

A robust and expected finding was the strong positive association between depressive and anxiety symptoms, reflecting the high comorbidity between these conditions in older adults [41,42,43]. This result reinforces the importance of adopting an integrated clinical perspective when assessing emotional functioning in later life, as co-occurring symptoms may have cumulative or interactive effects on functioning.

Age also emerged as a significant negative predictor of decision-making capacity, suggesting that increasing age is associated with reduced decisional abilities. This finding is consistent with literature indicating age-related declines in cognitive domains critical for decision-making, such as processing speed, executive functioning, and working memory [44,45,46]. Beyond cognitive decline, age-related factors such as reduced functional independence and lower engagement in cognitively stimulating activities may further contribute to diminished decisional capacity [47]. Nevertheless, it is important to emphasize that cognitive aging is heterogeneous, and protective factors such as cognitive reserve and continued engagement in meaningful activities may mitigate these effects [45].

Unexpectedly, education did not show a significant association with decision-making capacity, contrasting with previous findings linking higher literacy levels to better decision-making outcomes [48]. This discrepancy may be explained by the relatively high and homogeneous educational level of the sample, which may have limited variability and may have reduced the ability to detect significant effects.

Taken together, the findings of this study contribute to the existing literature by suggesting that depressive symptomatology may play a clinically relevant role in the capacity for decision-making in healthcare at higher symptom levels, even in cognitively healthy older adults, particularly when considered alongside other variables. The results also reinforce the central role of cognitive functioning while supporting a multidimensional perspective that integrates cognitive and affective factors.

From a clinical standpoint, and notwithstanding the preliminary status of the present findings concerning depression, these findings underscore the importance of incorporating both cognitive and emotional assessments into evaluations of decision-making capacity, in line with existing recommendations [10]. Specifically, assessing depressive symptomatology is crucial to avoid misinterpretations, particularly in cases where transient mood disturbances may affect performance without reflecting a stable cognitive decline. Given that depression is a potentially reversible condition, its identification and treatment should be followed by a reassessment of the capacity for decision-making to determine whether observed impairments persist. This approach supports more dynamic and ethically grounded assessment practices, promoting autonomy, self-determination, and dignity in older adults.

These findings can be situated within the four-abilities framework [8], which conceptualizes decisional capacity as comprising understanding, appreciation, reasoning, and expression of choice. The CAI-Health operationalizes each of these domains, and the present analyses concern the composite score. The possibility that age and affective symptoms bear differentially on individual abilities—depression having been proposed to affect appreciation in particular [11]—could not be examined here and warrants domain-level analysis in larger samples. Considered as a whole, the pattern observed reinforces the recommendation that capacity should be assessed multidimensionally rather than inferred from cognitive performance alone, since cognitive screening accounted for only a modest proportion of unique variance in decisional capacity in this sample.

The findings also support dynamic and person-centered approaches to capacity assessment. Best practice in this field increasingly emphasizes supported decision-making, in which the aim is to enable decisional capacity rather than to merely adjudicate it [49]. Practical elements include tailoring communication to the individual’s literacy and sensory profile, presenting information in stages and verifying comprehension, allowing sufficient time for deliberation rather than requiring immediate decisions, involving trusted supporters when the person so wishes, and drawing on multidisciplinary input where the clinical picture is complex.

A further consideration concerns how instruments of this kind should be positioned within capacity assessment. The CAI-Health employs a standardized hypothetical vignette, which affords comparability across individuals, a normative frame of reference, and the psychometric transparency that a defensible capacity determination requires. These are properties that decision-specific procedures cannot provide, and standardization should therefore be preserved. It should nonetheless be recognized that a vignette does not reproduce the emotional salience, time pressure, and interpersonal context of an actual healthcare decision. Performance on such an instrument should be understood as a structured indicator of decisional abilities rather than as a determination of capacity. Consistent with established professional guidance [7,10], the judgement of capacity remains a clinical judgement, grounded in standardized psychometric assessment and integrated with other sources of information—in particular a clinical interview with the person being assessed, together with collateral information from informants considered relevant to the specific decision at issue.

Finally, the present findings highlight the importance of addressing modifiable factors, such as depressive symptoms and cognitive functioning, as potential targets for intervention aimed at preserving decision-making capacity. Future research should adopt longitudinal and multidimensional approaches to further clarify the mechanisms underlying these relationships and to inform the development of targeted interventions that support decision-making in aging populations.

## 5. Conclusions

The present study contributes to the growing body of literature on the capacity for decision-making in healthcare by examining its associations with cognitive functioning, affective symptomatology, and sociodemographic factors in cognitively healthy older adults. The findings highlight that increasing age is robustly associated with reduced decision-making capacity, and that depressive symptomatology is also associated with it, although sensitivity analyses indicated that this latter association was confined to the small number of participants with clinically elevated symptoms. Together, these results reinforce the need to consider both cognitive and emotional dimensions when evaluating decisional abilities in later life. In contrast, anxiety symptoms did not emerge as a significant predictor, although their strong association with depressive symptoms confirms the well-established comorbidity between these conditions.

Overall, the results support a multidimensional understanding of the capacity for decision-making in healthcare, emphasizing that it is not solely determined by cognitive functioning but rather emerges from the dynamic interaction between cognitive, emotional, and demographic factors. These findings underscore the importance of adopting integrative and multidisciplinary approaches in both research and clinical practice, particularly in aging populations.

From a clinical and ethical perspective, this study reinforces the development of a systematic assessment of depressive symptoms alongside cognitive evaluation in capacity assessments. Given that depressive symptomatology may transiently impair decisional abilities without reflecting irreversible cognitive decline, its identification is critical to avoid misclassification of individuals as incapable. This highlights the importance of dynamic assessment processes, including reassessment following appropriate psychological or psychiatric intervention, to safeguard autonomy, self-determination, and dignity in older adults.

Several limitations should be acknowledged. The relatively small sample size (*N* = 60) may limit the statistical power and robustness of the findings. The ratio of cases to predictors in the full model falls below conventional recommendations and entails a risk of overfitting, as reflected in the difference between the unadjusted and the adjusted proportion of variance reported above; the reduced-model and case-exclusion analyses were conducted for this reason. The regression findings concerning depressive symptomatology proved sensitive to a small number of influential observations and should be regarded as provisional until replicated in samples with greater variability in affective symptoms. Additionally, the homogeneity of the sample, particularly in terms of sociodemographic characteristics, may have constrained variability and may have reduced the ability to detect associations between variables. The recruitment strategy might have contributed to this limitation. Participants were recruited through dissemination on social media and partnerships with institutions working with older adults. This approach is likely to have favored individuals who were healthier, more educated, more digitally literate, and more socially engaged than the general older adult population, and the resulting selection bias limits external validity. The educational distribution of the sample, in which slightly more than half had completed secondary or higher education, is consistent with this interpretation and may partly explain the absence of an association between education and decision-making capacity.

Furthermore, the cross-sectional design precludes causal inference. Although the term predictor is used here in its statistical sense, the associations reported describe covariation observed at a single point in time and do not establish that age or depressive symptoms produce changes in decisional capacity. Longitudinal designs are required to address questions of temporal ordering and causality.

Individuals with cognitive impairment were excluded to establish how decisional capacity varies within the range of normal cognitive aging, and to ensure that any observed effects could not be attributed to detectable cognitive decline. This choice, although methodologically justified, may have resulted in an underrepresentation of depressive and anxiety symptoms, given their higher prevalence in cognitively impaired populations [50]. Consequently, the findings related to affective symptomatology should be interpreted with caution. It is also important to consider that the length and cognitive demands of the evaluation protocol may have influenced participants’ performance, particularly among older individuals. It should further be acknowledged that the capacity for decision-making in healthcare is most often called into question clinically among individuals with mild cognitive impairment or dementia, and that the present findings do not extend to those populations; their clinical implications concern chiefly the interpretation of capacity in older adults whose cognitive screening results fall within normal limits.

Despite these limitations, this study offers several important contributions. It addresses a relatively underexplored domain by focusing on psychosocial predictors of the capacity for decision-making in healthcare and adopts an integrative approach that simultaneously considers cognitive, emotional, and sociodemographic variables. By doing so, it advances a more holistic and person-centered understanding of decisional capacity, grounded in both clinical and ethical considerations.

Future research should build upon these findings by employing larger and more diverse samples, allowing for greater statistical power and the exploration of subgroup differences. The inclusion of clinical populations, such as individuals with neurocognitive disorders or affective conditions, would provide a more comprehensive understanding of how these variables interact in contexts of increased vulnerability. Longitudinal designs would also be particularly valuable in clarifying causal relationships and temporal dynamics between cognitive decline, emotional states, and decision-making capacity.

Additionally, the development and use of shorter, psychometrically robust assessment protocols may help reduce participant burden and improve data quality. Further research should also aim to refine theoretical models that integrate cognitive and affective processes in decision-making, contributing to more precise and clinically useful frameworks.

Finally, these findings have practical implications for healthcare practice. Enhancing health literacy among older adults may support more effective decision-making, particularly in complex or unfamiliar clinical situations [51]. Healthcare professionals should adapt communication strategies, ensure adequate time for reflection, and verify patients’ understanding of relevant information to support informed decision-making. Such approaches are essential to promoting autonomy and ensuring ethically sound, person-centered care in aging populations.

## Figures and Tables

**Table 1:** Descriptive Statistics for the Study Variables (*N* = 60).

Variable	Mean (*SD*)	Minimum	Maximum
CAI-Health	27.10 (4.16)	15	34
MoCA	23.65 (2.84)	17	30
GAI	5.37 (5.77)	0	20
GDS	5.47 (5.16)	0	24

**Table 2:** Spearman’s correlation between the capacity for decision-making in healthcare, depressive symptoms, anxiety symptoms, and cognitive performance (*N* = 60).

	CAI-Health	GDS	GAI	MoCA
CAI-Health	1.00			
GDS	−0.076	1.00		
GAI	−0.007	0.730 **	1.00	
MoCA	0.480 **	−0.108	−0.095	1.00

** *p* < 0.001.

**Table 3:** Results of the multiple linear regression analysis (*N* = 60).

	*B*	*SE B*	95% CI	β	*p*	VIF
GDS	−0.331	0.157	[−0.646, −0.016]	−0.411	0.040	3.59
GAI	0.115	0.140	[−0.166, 0.396]	0.159	0.415	3.55
Age	−0.342	0.082	[−0.507, −0.177]	−0.490	<0.001	1.31
MoCA	0.177	0.182	[−0.188, 0.542]	0.121	0.336	1.46
**Education**						
Primary Education vs. 3rd level of Basic Education	−0.984	1.329	[−3.650, 1.682]	−0.095	0.462	1.57
Primary Education vs. Secondary Education	0.271	1.242	[−2.220, 2.763]	0.031	0.828	1.85
Primary Education vs. Higher Education	1.795	1.370	[−0.955, 4.545]	0.174	0.196	1.67

*R*^2^ = 0.449, adjusted *R*^2^ = 0.375, *F*(7, 52) = 6.053, *p* < 0.001.

**Table 4:** Sensitivity analyses for the regression of the capacity for decision-making in healthcare.

Model Specification	*B* Age	*p*	*B* GDS	*p*	*R* ^2^
Full model (*N* = 60)	−0.342	<0.001	−0.331	0.040	0.449
Full model, influential cases excluded (*n* = 57)	−0.290	0.001	+0.012	0.951	0.406
Reduced model (age, MoCA, GDS, GAI)	−0.323	<0.001	−0.390	0.015	0.405
Reduced model, influential cases excluded (*n* = 57)	−0.282	0.001	−0.086	0.665	0.330

Influential cases are the three participants with GDS-30 scores of 20 or above, identified through Cook’s distance, leverage and standardized residuals.

## Data Availability

The data presented in this study are available on request from the corresponding author. The data are not publicly available due to privacy and ethical restrictions.
